# miRNAs Signature in Head and Neck Squamous Cell Carcinoma Metastasis: A Literature Review

**Published:** 2016-06

**Authors:** Soussan Irani

**Affiliations:** 1 Dental Research Center, Dept. of Oral and Maxillofacial Pathology, School of Dentistry, Hamadan University of Medical Sciences, Hamadan, Iran. and Lecturer at Griffith University, Gold Coast, Australia.

**Keywords:** Head and Neck Cancer, Metastasis, miRNA

## Abstract

**Statement of the Problem:**

Head and neck cancers include epithelial tumors arising in the oral cavity, pharynx, larynx, paranasal sinuses, and nasal cavity. Metastasis is a hallmark of cancer. MicroRNAs (miRNAs) are endogenous small non-coding RNAs involved in cell proliferation, development, differentiation and metastasis. It is believed that miRNA alterations correlate with initiation and progression of cancer cell proliferation or inhibition of tumorigenesis. Moreover, miRNAs have different roles in development, progression, and metastasis of head and neck squamous cell carcinoma (HNSCC). Altered expression of miRNAs could be novel molecular biomarkers for the definite diagnosis of cancer, metastatic site, cancer stage, and its progression.

**Purpose:**

The purpose of this review was to provide a comprehensive literature review of the role of miRNAs in head and neck cancer metastasis.

**Search strategy:**

A relevant English literature search in PubMed, ScienceDirect, and Google Scholar was performed. The keywords ‘miRNA’, ‘head and neck’, and ‘cancer’ were searched in title and abstract of publications; limited from 1990 to 2015. The inclusion criterion was the role of miRNAs in cancer metastasis. The exclusion criterion was the other functions of miRNAs in cancers. Out of 15221 articles, the full texts of 442 articles were retrieved and only 133 articles met the inclusion criteria.

**Conclusion:**

Despite the advances in cancer treatment, the mortality rate of HNSCC is still high. The potential application of miRNAs for cancer therapy has been demonstrated in many studies; miRNAs function as either tumor suppressor or oncogene. The recognition of metastamir and their targets may lead to better understanding of HNSCC oncogenesis, and consequently, development of new therapeutic strategies which is a necessity in cancer treatment. Development of therapeutic agents based on miRNAs is a promising target.

## Introduction

Head and neck cancer is the sixth most common malignancy, comprising 6% of all cancers[1] and includes epithelial tumors arising in the oral cavity, pharynx, larynx, paranasal sinuses, and nasal cavity. Almost all of these malignancies are squamous cell carcinoma (SCC).[2] Head and neck squamous cell carcinomas (HNSCCs) are highly heterogeneous cancers. Oral squamous cell carcinoma (OSCC) is the most frequently occurring cancer in the head and neck area (90% of all cases). Metastasis is the main cause of death[3] and mostly arises in the tongue, floor of mouth, gingiva, and buccal mucosa.[4]

Metastasis is a hallmark of cancer and is defined as the transfer of disease from one organ or part of an organ to another part that is not directly connected to it. Tumor cells penetrate into vascular or lymphatic channels and provide the opportunity to spread. The spread is dictated by local anatomy, and each site has its own pattern.[5] The process of metastasis is complex and involves sequential steps. First, the cells detach from the primary site. Then, they spread in the tissue, move away through the extracellular matrix, invade blood vessels, and settle in the microvasculature. Finally, the cells extravasate through the vessel wall and proliferate in the recipient tissue.[6] Cervical lymph node metastasis is the strongest determinant of patient prognosis in HNSCCs[7] which decreases the survival rate by about 50%.[8-9] HNSCC is not usually detected in the early stages of the disease due to the absence of clinical symptoms.[10]

MicroRNAs (miRNAs) are endogenous small non-coding RNAs; they can regulate gene expression in the post–transcriptional stage by interacting with the 3' untranslated region (3' UTR) of the target mRNA.[11]Several miRNAs regulate cancers; miRNAs play crucial roles in proliferation, differentiation, apoptosis, survival, motility, invasion and metastasis, and morphogenesis.[12] It has been shown miRNAs can be used as novel molecular biomarkers for cancer diagnosis.[13] For example, circulating miRNAs are associated with distinct metastatic site; therefore, they are powerful tools to evaluate the disease stage and progression.[14]

The purpose of this review was to provide a comprehensive literature review of the role of miRNAs in head and neck cancer metastasis.

**Searching Strategy**

A relevant English literature search in PubMed, ScienceDirect, and Google Scholar was performed. The keywords ‘miRNA’ ‘head and neck’ and ‘cancer’ were searched in title and abstract of publications; limited to 1990 to 2015. The inclusion criterion was the role of miRNAs in cancer metastasis. The exclusion criterion was the other functions of miRNAs in cancers. A total of 15221 articles were found. Among them, the full text of 442 articles were retrieved and recited. Only 133 articles met the specific inclusion criteria for this review.

**miRNAs biology**

In human, miRNAs have a stem-loop structure which are capped at the 5΄-end and have a 3΄-poly (A).[15]Functional miRNA is produced through a two-step transcript maturation process. The first step occurs in the nucleous, which is facilitated by an endogenous RNase III called Drosha, and the double-stranded RNA binding domain protein DGCR8.[16] After cleavage of both strands of the pri-miRNA transcript by Drosha, a stem-loop precursor molecule (pre-miRNA) is produced.[17] Pre-miRNA molecules are then transported to the cytoplasm.[18] Finally, both strands of the pre-miRNA are cut at the base of stem-loop by the RNA III enzyme Dicer and dsRBD protein TRBP. The final product is a 22-nucleotide long molecule.[19]

**miRNAs regulate the target genes**

Literature shows miRNA and its target mRNA have interaction in the 5΄-end of the miRNA. For target sequence recognition, the sequence complementarity between nucleotides 2-8 is essential.[20] The 3΄-UTR region of the target mRNA called “seed region” in the 5΄-end of the miRNA determines the mechanism by which the miRNA regulates the target.[21] Down-regulation of target genes happens at the protein level but the regulation occurs at the translational level; therefore, the mRNA level remains unaffected.[22] The mechanism by which miRNAs regulate mRNA depends on the degree of miRNA complementarity with the mRNA molecule. Identification of miRNA targets is difficult because only 6–8 bases align perfectly with the target mRNA’s 3΄-UTR region.[23] The predicted targets for the differentially expressed miRNAs are significantly enriched for protein-coding tumor suppressors and oncogenes. A number of the predicted targets, along with the tumor suppressors and oncogenes have been confirmed experimentally. For instance, elevated expression level of miR-155 in HNSCC may explain its possible role in oral carcinogenesis[24] by targeting tumor suppressors such as APC.[25]

**The role of miRNAs in regulating** **EMT/MET**

Invasion is the initial step in the metastatic process. The mode of tumor invasion is the most significant prognostic factor for the presence of lymph node metastasis. Cases with grades 1-3 mode of invasion show a low frequency of metastasis; while, those cases with grade 4 mode of invasion show a high frequency of metastasis.[26] Epithelial-mesenchymal transition (EMT) is characterized by down-regulation of E-cadherin a cell adhesion molecule, and acquisition of mesenchymal markers such as N-cadherin, vimentin and fibronectin to gain cell motility and invasiveness.[27] Both EMT and Mesenchymal-epithelial transition (MET) are essential for cancer progress, as EMT of primary tumor cells is a necessity for motility and invasiveness. In addition, MET is important for the final stage of metastasis when the extravasated cancer cells revert to epithelial cells and proliferate as a secondary tumor in the metastatic site.[28] Some transcription factors are the inducers of EMT and tumor metastasis including, Snail, Slug, Twist, ZEB1, and ZEB2.[29] Several miRNAs inhibit EMT such as miR-573,[30] and miR-33b.[31] Overexpression of miR-7 also suppresses migration and invasion and partially reverses EMT via targeting IGF1R in gastric cancer. Additionally, overexpression of miR-7 reduces metastasis.[32] However, some miRNAs such as miR-214,[33] and miR-544a promote EMT.[34]

**The role of miRNAs in angiogenesis in cancers**

Angiogenesis has an essential role in tumor progression and metastasis. Vascular endothelial growth factor (VEGF) is one of the most important factors in angiogenesis and lymphangiogenesis.[35-36] VEGF-A expression level is conversely related to the miR-126 expression level in cancers such as lung cancer.[37] The miR-17-92 cluster is positively associated with angiogenesis in some cancers; however, individual components of the miR-17-92 cluster have opposite effects on angiogenesis.[38-39]

**The role of miRNAs in cell proliferation and apoptosis**

MicroRNA alterations correlate with the initiation and progression of cancer cell proliferation or inhibition of tumorigenesis. For example, miR-155 is up-regulated in OSCC and its dysregulation is associated with the low level of CDC73, a tumor suppressor gene; therefore, it promotes cancer cell proliferation.[40] A previous study on OSCC cells indicated that transfecting with miR-125b or miR-100 significantly decreased cell proliferation; however, co-transfection had a greater effect on proliferation than individual transfection.[41] miR-128 also acts as a tumor suppressor and inhibits the growth of HNSCC cells.[42] Down regulation of miR-29 in OSCC[43] is a positive regulator of p53.[44] miR-15 and miR-16 are the regulators of Bcl-2, an anti-apoptotic factor. Therefore, endogenous levels of miR-15 and miR-16 correlate inversely with the Bcl-2 protein level.[45] In an *in vitro* study, miR-375 induced apoptosis in HNSCC by targeting TNF-α.[46]

**The role of miRNAs in cancer development and metastasis**

Previous studies have indicated that dysregulation of miRNAs plays a crucial role in the progression of oral precancerous lesion from dysplasia to OSCC. For instance, miR-31 negatively controls oral leukoplakia progression through the regulation of fibroblast growth factor 3 (FGF3). On the contrary, miR-21, miR-181b, and miR-345 are up-regulated in oral dysplasia and associated with lesion severity.[47] Overexpression or subexpression of miRNAs in cancer has the ability to activate or block the target mRNAs. In a metastatic carcinoma, the miRNA expression profile has been shown to be different from a non-metastatic tumor.[48] Therefore, interference with the expression level of miRNAs has an effect on cancer prognosis. This finding provides a therapeutic use for miRNAs.[49]

Regarding the role of miRNAs in cancer, they are divided into two main groups of tumor suppressor and oncogenes (oncomirs). Altered miRNA expression depends on its role as a tumor suppressor or oncogene.[50] Furthermore, tumor suppressor miRNAs have been shown to be down-regulated in cancers; whereas, oncogene miRNAs are up-regulated.[51] Additionally, metastamir, a specialized family of miRNAs, has pro- and anti-metastatic effects.[52]

**Tumor suppressor miRNAs involved in HNSCC metastasis**

The literature review shows miR-1 is down-regulated in hypopharyngeal SCC. It has been shown miR-1 can suppress metastasis in HNSCC by targeting TAGLN2, a gene which mediates cell migration and invasion.[53] Let-7d, a member of the let-7 family of miRNAs acts as a tumor suppressor, most likely via targeting RAS.[54] A reduction of let-7d expression in OSCC tissues has been reported. Let-7d was negatively correlated to Twist, Snail, and EMT transcription markers in OSCC cell lines. In addition, let-7d was significantly decreased in regional metastatic lymph nodes of OSCC patients.[55] Combined low levels of miR-205, and let-7d expression are associated with distant metastasis.[56] Moreover, decreased let-7d expression in HNSCC is associated with poor prognosis. Both miR-17 and miR-20a are down-regulated in advanced migratory OSCC, and their expression levels are negatively controlled by advanced TNM stage and lymph node metastasis. ITGβ8 is a direct target of both miR-17 and miR-20a. Thus, they can be used as prognostic biomarkers for OSCC.[57] The miR-29 family is significantly down-regulated in HNSCC tissues and cell lines suggesting their contribution to metastasis in HNSCC through laminin γ2 (LAMC2) and α6 integrin (ITGA6).[58] A previous study demonstrated that the transforming growth factor beta (TGF-β) signaling which contributes to EMT process,[59] inhibits the expression of miR-29 family, and promotes the expression of extracellular matrix (ECM) components.[60] miR-34b plays an important role in EMT by targeting Snail.[61] In HNSCC, up-regulation of miR-34 b/c has been reported.[62-63] but the target gene has not yet been identified.[63] In addition, ectopic transfection of miR-99 family (miR-100) in HNSCC cell lines decreases cell migration, suggesting the role of miR-99 family as a tumor suppressor by targeting IGF1R and mTOR signaling pathways.[64] miR-125b has also been shown to have a role in tumor suppression by inhibiting cancer cell proliferation, migration, and invasion in skin SCC by down-regulation of matrix metalloproteinase -13 (MMP13).[65] miR-125b also significantly decreases the proliferation of OSCC cells.[41] miR-126 is a negative regulator of VEGF-A and induces nodal metastasis in OSCC patients. Therefore, downregulation of miR-126 induces angiogenesis and lymphangiogenesis by targeting VEGF-A, and basic fibroblast growth factor (bFGF).[66] miR-133a functions as a tumor suppressor in HNSCC and is significantly down-regulated in HNSCC tissues. MSN, an actin binding protein, involved in cell motility, is a novel target of miR-133a.[67] miR-138, a multi-functional molecule regulator, is frequently down-regulated in OSCC, which enhances cancer cell proliferation and invasion by targeting GNA12 mRNA, a proto-oncogene.[68] miR-138 is a tumor suppressor in HNSCC cells by suppressing invasion and promoting apoptosis.[69] In addition, miR-138 regulates cell migration and invasion in HNSCC cells by targeting RhoC and ROCK2.[70] The reduction of miR-138 in highly metastatic OSCCs is associated with elevated expression of RhoC and ROCK2 which enhances the activity of the Rho GTPase signaling cascade to increase metastasis.[71] miR-153 prevents EMT, returns the mesenchymal phenotype (MET phenomenon) of cells to epithelial-like cells, and decreases cellular invasive ability. Low expression level of miR-153 is related to OSCC metastasis by targeting SNAI1 and ZEB2.[72] The miR-200 family can inhibit EMT and tumor cell migration by targeting ZEB1/ZEB2.[73] Decreased expression level of miR-200c has been shown in the regional lymph nodes of HNSCC patients, and is associated with increased expression of BMI1 in primary tumors.[74] miR-222 acts as a tumor suppressor in OSCC and inhibits tumor invasion and metastasis by indirectly regulating MMP1 expression via targeting SOD2 mRNA (superoxide dismutase 2).[75] In HNSCC, miR-363 inhibits metastasis by down-regulation of podoplanin (PDPN), a proto-oncogene.[76] A low miR-375 expression level is associated with poor prognosis and distant metastasis in patients with HNSCC.[77] In addition, low expression level of miR-375 in the late stage of cancer is correlated with increased invasive capabilities and metastasis of OSCC cells.[78] Table 1 summarizes the role of tumor suppressor miRNAs in HNSCC metastasis.

**Table 1:** Summary of Suppressor miRNAs and proposed target genes

**Dysregulated miRNA (Reference)**	**Expression status**	**Proposed target gene**
miR-1 [53] let-7d [55] miR-17 and miR-20a [57] miR-29 [58-59] miR-34 [63] miR-99 family (miR-100 and miR-99a) [64] miR-125b [41] miR-126 [66] miR-133a [67] miR-138 [70-71] miR-153 [72] miR-200 [73] miR-200c [74] miR-222 [75] miR-363 [77] miR-375 [1]	Underexpression Underexpression Underexpression Underexpression Overexpression Underexpression Underexpression Underexpression Underexpression Underexpression Underexpression Underexpression Underexpression Underexpression Underexpression Underexpression	TAGLN2 Twist, Snail ITGβ8 LAMC2, ITGA6, TGF-β Unknown IGFIR, mTOR KLF13, CXCL1 and FOXA1 VEGF-A, bFGF MSN GNA12, RhoC and ROCK2 SNAI1 and ZEB2 ZEB1,ZEB2 BMI1/ZEB1 MMP-1 Podoplanin(PDPN) Unknown

**OncomiRs involved in HNSCC metastasis**

It is reported that miR-10b is down-regulated in HNSCC cell lines and induces cell proliferation.[79] miR-10b significantly increases oral cancer cell migration and invasion.[80] Recently, miR-21 has been studied more frequently as a prognostic marker of HNSCC. It is stated that miR-21 down-regulates programmed cell death 4 (PDCD4) in OSCC.[81] miR-21 up-regulation is correlated with advanced stages of OSCC and lymph node metastasis.[82] Therefore, a positive relationship exists between high expression of miR-21 and worsened prognosis in HNSCC. In addition, increased expression level of miR-21 in tumor stroma is significantly related to worsened outcome.[83] miR-31 is up-regulated in HNSCC and activates hypoxia-inducible factor-1α (HIF-1α) in normoxia and enhances the oncogenesis via factor inhibiting HIF-1(FIH). In fact, miR-31 functions as an oncogene only when oxygen supply is sufficient. It is believed that miR-31 also promotes some events of early stages of OSCC such as proliferation and tumorigenicity.[84] In addition, miR-31 is significantly expressed in early stage of OSSC tissue samples and its expression is associated with the lack of lymph node metastasis.[78] Increased expression level of miR-93 is associated with HNSCC lymph node metastasis.[85] In other cancer types, miR-93 promotes tumor invasion and metastasis through regulating EMT, angiogenesis, and disrupting adhesion between cancer cells and extracellular matrix (ECM).[86-87] However, the target gene of miR-93 in HNSCC is not elucidated. High expression of miR-134 is associated with nodal metastasis and mortality and miR-134 functions via targeting WW domain-containing oxidoreductase (WWOX) gene, a tumor suppressor.[88] In addition, up-regulation of miR-155 in OSCC is correlated with the histologic grade and can be used as a potential prognostic biomarker.[89] miR-155-5p induces metastasis and is associated with poor prognosis; miR-155-5p inhibitor down-regulates signal transducer and activator of transcription 3 (STAT3) by activating *suppressor of cytokine signaling 1* (SOCS1).[90] miR-181b is highly expressed in OSCC patients with lymph node metastasis.[91] According to a previous study, miR-181 was up-regulated, during progression of oral leukoplakia to dysplasia and finally to invasive carcinoma.[47] In HNSCC, miR-211 promotes tumor invasion and metastasis by targeting transforming growth factor β type II receptor (TGFβRII). There is an inverse correlation between miR-211 and TGFβRII expression during HNSCC metastasis.[92] A previous study indicated that miR-211 was significantly expressed in OSCC tissue samples with N2 nodal metastasis and vascular invasion compared with non-cancerous tissues.[93] miR-223 plays crucial role in tumourigenesis and its function is cancer type-specific. Reduced level of miR-223 has been observed in hematopoietic malignancies; however, elevated expression of miR-223 is indicated in several types of solid tumors of head and neck area.[64] Table 2 summarizes the role of oncomiRs in HNSCC metastasis. 

**Table 2:** Summary of oncogene miRNAs and the proposed target genes

**Dysregulated** **miRNA (Reference)**	**Expression status**	**Proposed** **Target gene**
miR-10b [80] miR-21 [81] miR-31 [84] miR-93 [85] miR-134 [88] miR -155 -5p [90] miR-181b [91] miR-211 [92] miR-223 [63]	Underexpression Overexpression Overexpression Overexpression Overexpression Overexpression Overexpression Overexpression Overexpression	Unknown PDCD4 FIH Unknown WWOX STAT3 Unknown TGFβRII Unknown

**The role of miRNAs in diagnosis and prognosis of cancer**

It has been shown that miRNAs can be a non-invasive diagnostic tool in detecting HNSCC and determining prognosis as they can be detected in saliva. For example, miR-125a and miR-200a are under-expressed in saliva of OSCC patients compared to healthy controls.[94] Besides, a previous study showed that the miRNA expression profile differs in different sites of the head and neck area and reflects the developmental origin of tissue; moreover, miRNAs can distinguish the cancers from benign tumors, non-neoplastic lesions and normal tissue. For instance, the expression ratio of miR-221 to miR-375 can be used to distinguish tumor from normal tissue with high specificity and sensitivity[95] and altered expression level of 22 miRNAs can accurately distinguish chronic pancreatitis from pancreatic cancer.[96] Besides, the different expression profile of miRNAs in esophageal squamous cell carcinoma is a diagnostic and prognostic factor as well as a tool for distinguishing cancer from normal tissue.[97] Moreover, miRNAs help to predict the primary site of metastatic disease.[11] In head and neck area, the primary site is unknown in up to 10% of metastatic squamous cell carcinomas. By knowing the origin of the disease, specific therapeutic regimens such as reducing the radiation field can be employed, which consequently minimizes the morbidity.[98] Interestingly, the miRNA expression level in primary tumors and their corresponding metastatic sites remains consistent in different patients; however, it differs in the same tumor in different locations. All together, the miRNA expression profile can be used as a diagnostic tool to distinguish the cancers from benign tumors and other benign conditions and also from normal tissues, as well as to predict the origin of unknown primary tumors in the head and neck area.[98] Decreased expression levels of miR-205 and let-7d are significantly associated with higher chances of loco-regional recurrence and shorter survival in patients with HNSCC.[56] High expression level of miR-205 can be used to detect HNSCC lymph node metastasis.[99] A previous study identified lower expression level of miR-451 in HNSCC tumors as a strong predictor of recurrence of the tumor.[100] Moreover, in some cancers such as lung cancer, existence of cancer-specific miRNAs can diagnose the cancer and classify the tumor subtype.[101] 

Additionally, miRNAs may have an impact on the analysis of surgical margins for residual cancer extension.[102] High endogenous expression of mir-205 in normal and HNSCC cells and its low expression in lymph node tissue makes it a worthy biomarker for detecting a small number of tumor cells in the metastatic site.[99] Taken together, these findings show that altered expression of miRNAs could be a promising marker for HNSCC prognosis. 

## Discussion

Due to a gradual increase in the number of cancer patients, careful evaluation of patients has a great impact on decreasing the cancer-related morbidity and mortality rate. Among the cancer groups, HNSCCs consist of highly heterogeneous lesions.[103]

It seems that in the metastatic phenotype of miRNAs, a global reduction of miRNA abundance plays a causal role;[11] however; several miRNAs are up-regulated in specific tumors. Moreover, miRNAs act as either oncogenes or tumor suppressors which make them prospective targets for therapeutic development against cancers.[104]

There are many challenges in the application of miRNA-based therapies.[32] For instance, miR-1 suppresses metastasis in HNSCC by targeting TAGLN2;[53] however, targeting MET, a proto-oncogene, is the underlying mechanism for anti-tumorigenic property of miR-1 in lung cancer. 

Similarly, miR-1 down-regulation was observed in other types of cancer such as lung cancer.[105] A previous study indicated that miR-29 was down-regulated in breast cancer; miR-29 has an essential role in inhibiting growth of breast cancer cells via down-regulation of B-Myb.[106] But in HNSCC tissue, the target genes of mir-29 are LAMC2 and ITGA6.[58] The miR-34 family may inhibit invasion and metastasis in different cancer types. For example, miR-34a and b are suggested as tumor suppressors in several cancers such as gastric cancer,[107] breast cancer,[108] and hepatocellular carcinoma mainly by targeting p53.[109] mir34a/c may function as a metastasis suppressor in breast cancer through targeting Fos-related antigen 1 (FOSL1).[110] Nonetheless, the target gene of miR-34 in HNSCC is not clear.[63] miR-133a acts as a tumor suppressor in esophageal squamous cell carcinoma by targeting Fascin Actin-Bundling Protein 1 (FSCN1), an actin bundling protein which promotes the degradation of ECM, and matrix metalloproteinase 14 (MMP14).[111] But the target gene of miR-133a in HNSCC is MSN.[67] miR-99 functions as a tumor suppressor in some cancers via different targets. For example in HNSCC, it acts via IGFIR and mTOR, though in cervical cancer the target gene is Tribbles (TRIB2) which controls the specificity of the activation of mitogen-activated protein kinases (MAPK).[112] miR-138 also targets different genes in human colorectal cancer (Twist 2) compared with HNSCC (GNAI2, RhoC, and ROCK2).[113] miR-10b was the first link between metastasis and miRNAs indicated in breast cancer. Twist which induces EMT is a target of miR-10b.[114] Moreover, miR-10b targets E-cadherin and modulates metastasis in breast cancer;[115] however; its target gene in HNSCC is still unknown. The literature shows that miR-21 is one of the most studied miRNAs.[116] Although miR-21 functions as an oncogene via programmed cell death 4(PDCD4) in HNSCC,[81] and esophageal carcinoma,[117] it stimulates cell invasion and promotes metastasis in breast cancer by targeting both PDCD4, and tropomyosin 1 (TPM1), a tumor suppressor gene.[118-119] On the other hand, miR-21 controls esophageal squamous cell carcinoma invasion by targeting the tissue inhibitor of matrix metalloproteinase-3 (TIMP3) molecules which control ECM.[120] miRNAs expression pattern and function are cancer-type-specific. For example, up-regulation of miR-31 has been reported in colorectal cancer;[121] however, reduced level of miR-31 was observed in breast cancer.[122] While it inhibits metastasis in breast cancer,[123] it induces invasion and metastasis in colon cancer via TGF-β.[124] In addition, miR-181a and mir-181b suppress glioma tumor growth,[125] but contributes to tumor progression in thyroid papillary carcinoma.[126] miR-211 acts as a tumor suppressor in melanoma,[127] whereas; it functions as an oncomir in colon cancer.[128] Up-regulation of miR-222 in gastric carcinoma is associated with lymph node metastasis, vascular invasion and TNM stage,[129] but miR-222 regulates OSCC as a tumor suppressor and inhibits tumor invasion and metastasis.[12] MiR-223 acts as an oncogene in gastric cancer, and enhances cancer invasion and metastasis by targeting tumor suppressor genes F-box and WD40 domain protein 7 (FBXW7) and erythrocyte membrane protein band 4.1-like 3 (EPB41L3). But, the target gene of miR-223 in HNSCC is not clear.[63] miR-363 is a tumor suppressor in T cell lymphoma,[130] however; it acts as an oncogene in breast cancer.[131]

## Conclusion

In conclusion, despite advances in cancer treatment, the mortality rate of HNSCC is still high. The potential application of micro-RNAs (miRNAs) in cancer therapy has been demonstrated in many studies; miRNAs function as either tumor suppressor or oncogene. The recognition of metastamir and their targets may lead to better understanding of HNSCC oncogenesis, and consequently the development of new therapeutic strategies for HNSCC which is a necessity in cancer treatment. Development of therapeutic agents based on miRNAs is a promising target. 
